# Five-year follow-up of phase II trial of autologous cord blood mononuclear cells for bronchopulmonary dysplasia prevention in very preterm infants

**DOI:** 10.1093/stcltm/szag045

**Published:** 2026-07-28

**Authors:** Zhuxiao Ren, Jiangxue Han, Qi Zhang, Wei Wei, Yongsheng Li, Shumei Yang, Fang Xu, Chuan Nie

**Affiliations:** Department of Neonatology, Guangdong Women and Children Hospital, Guangdong Provincial Quality Control Center for Neonatal Intensive Care Medicine, Southern University of Science and Technology, Guangzhou, 511442, China; Department of Ultrasonography, Children’s Hospital, Zhejiang University School of Medicine, National Clinical Research Center for Child Health, Hangzhou, 310052, China; Department of Neonatology, Guangdong Women and Children Hospital, Guangdong Provincial Quality Control Center for Neonatal Intensive Care Medicine, Southern University of Science and Technology, Guangzhou, 511442, China; Department of Cord blood, Guang Dong Cord Blood Bank, Guangzhou, 511440, China; Department of Cord blood, Guang Dong Cord Blood Bank, Guangzhou, 511440, China; Department of Neonatology, Guangdong Women and Children Hospital, Guangdong Provincial Quality Control Center for Neonatal Intensive Care Medicine, Southern University of Science and Technology, Guangzhou, 511442, China; Department of Neonatology, Guangdong Women and Children Hospital, Guangdong Provincial Quality Control Center for Neonatal Intensive Care Medicine, Southern University of Science and Technology, Guangzhou, 511442, China; Department of Neonatology, Guangdong Women and Children Hospital, Guangdong Provincial Quality Control Center for Neonatal Intensive Care Medicine, Southern University of Science and Technology, Guangzhou, 511442, China

**Keywords:** neurodevelopment, bronchopulmonary dysplasia, very preterm neonates, autologous cord blood mononuclear cells, follow-up

## Abstract

**Background:**

We previously performed a phase II clinical trial involving the intravenous infusion of autologous cord blood mononuclear cells (ACBMNCs) to prevent bronchopulmonary dysplasia in premature infants. We reported the outcomes before discharge and at 2-year follow-up of the enrolled infants. We have reported the developmental outcomes at the 5-year follow-up visit.

**Methods:**

We evaluated the 5-year long-term outcomes of ACBMNCs infusion, including growth, respiratory, and neurodevelopmental outcomes, by interviewing the parents. Children’s development was assessed using scores on the Chinese version of the Ages & Stages Questionnaires (ASQ), third edition (ASQ-3), completed by the parents or guardians. The primary outcome was the neurodevelopmental outcome. The secondary outcomes were growth and respiratory outcomes.

**Results:**

In terms of the primary endpoint, intravenously infused ACBMNCs significantly improved the scores of the fine motor and personal-social domains in the ASQ (*P* = .044 and *P* = .048, respectively). This study also showed high consistency between the motor index in the Bayley score at a 2-year follow-up with both gross and fine motor domain scores, as well as the language index with communication, problem-solving, and personal-social domain scores. Although pneumonia-related hospitalizations were similar between the two groups, the intervention group showed a lower possibility of nocturnal cough.

**Conclusion:**

Infants who received intravenous ACBMNC infusion had better fine motor and personal-social abilities at 5 years than those in the control group. In addition to providing reassuring safety data, the study suggested longer-term neurodevelopmental benefits of ACBMNC intervention early in the NICU course. Further randomized placebo-controlled trials are essential to confirm these findings.

**Trial registration:**

The trial was prospectively registered at ClinicalTrials.gov (NCT02999373), registered on December 21, 2016.

## Graphical Abstract

**Graphical Abstract:**
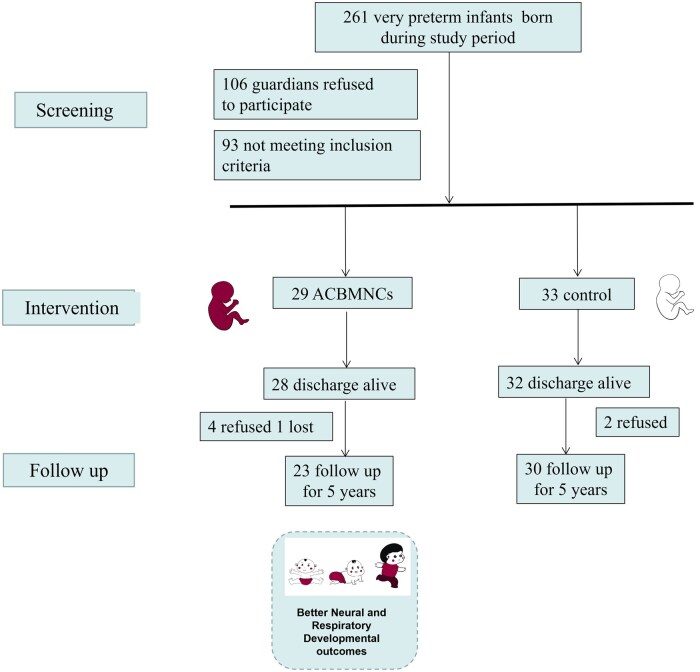

Significance statement Infants who received intravenous autologous cord blood mononuclear cells (ACBMNCs) infusion had better fine-motor and personal-social abilities at 5 years than those in the control group. In addition to providing reassuring safety data, the study suggested longer-term neurodevelopmental benefits from ACBMNCs intervention early in the NICU course. Further large-scale, randomized, placebo-controlled trials are needed to validate the present findings.

## Introduction

Bronchopulmonary dysplasia (BPD) is a severe complication of preterm birth.^1^ BPD is caused by alveolar simplification and dysregulated vascularization.^1^^,^^2^ Severe BPD remains an important cause of impaired lung function and neurodevelopmental delay in survivors.^3^ Early childhood behavioral outcomes may be determinants of mental health later in life, which raises the potential for early interventions.^3^^,^^4^ To date, there are few effective treatments for improving the long-term outcomes of BPD in clinical practice.^1^^,^^3^

Mononuclear cells (MNCs) are the main sources of stem and progenitor cells in the cord blood.^5^ MNC is mainly composed of mesenchymal stem cells (MSCs) and hematopoietic stem cells.^6^ In recent years, stem cells have shown great potential for the prevention and treatment of BPD.^7–11^ A few phase I and II clinical trials have reported the safety, feasibility, and effectiveness of stem cells in alleviating BPD severity in very preterm infants at high risk of developing BPD.^12–17^ Significant differences in cognitive, language, and motor skills are most commonly observed in patients with higher BPD grades.^3^^,^^18^ Park and coworkers showed no significant benefit in either respiratory outcomes or behavior profiles in extremely preterm infants intratracheally transfused with exogenous MSCs intratracheally at a 5-year follow-up.^19^ Our previous clinical study showed that autologous cord blood mononuclear cells (ACBMNCs) reduced pneumonia-related hospitalizations and improved neurodevelopmental outcomes during a 2-year follow-up.^20^ One rare study investigated long-term neurodevelopmental outcomes and whether neurodevelopmental improvement persisted with age in infants who received stem cell intervention. In this prospective study, we analyzed the neurodevelopmental and respiratory outcomes of very preterm infants enrolled in a phase II study of ACBMNCs infusion to prevent BPD at 5-year follow-up.

## Methods

### Study design and participants

This was a prospective follow-up study of participants enrolled in a double-blind, nonrandomized, placebo-controlled phase II clinical trial of a single intravenous infusion of ACBMNCs or normal saline placebo in very preterm infants at less than 32 gestational weeks. As previously reported, 62 infants were enrolled: 29 in the intervention group and 33 in the control group.^20^ The parents, hospital, research staff, and medical personnel directly involved in patient care and follow-up were blinded to the group allocation throughout the study period. Details of the interventions have been reported previously.^20^ Briefly, a single dose of ACBMNCs (5 × 10^7^ cells/kg) or normal saline was intravenously administered within 24 h of birth. The 2-year follow-up study reported that the stem cell intervention group showed fewer pneumonia-related hospitalizations and neurodevelopmental delays (assessed by the Bayley score). All living infants enrolled in the previous trial were contacted via telephone. Five infants in the intervention group and 2 in the control group were lost to follow-up. As the original study was double-blind, we maintained a double-blind design during the follow-up period.

### Procedures of study

#### Basic characters

The gender, gestational age (GA), birth weight, birth length, main caregivers, education level, rate of parents as main caregivers, and smoking in main caregivers were collected through telephone interviews.

#### Anthropometric characteristics

Patient height and body weight were determined for growth assessment. Weight and height were reported by the parents if these parameters were measured during the latest month before the interview; otherwise, they were referred to the clinic for height and body weight measurements. The weight and height percentiles of age- and sex-matched children were determined using the 2009 Chinese National Growth Charts.^21^ Low body weight was defined as a body weight of less than 3% of age- and sex-matched children. Short stature was defined as a body length shorter than 3% in age- and sex-matched children. Body mass index (BMI) was calculated as weight (kg) divided by height squared (cm).

#### Respiratory outcomes

The occurrence of wheezing, nocturnal cough, supplemental oxygen requirement, and rehospitalization due to pneumonia during the previous year was documented by interviewing the main caregivers and checking the hospital records.

#### Neurodevelopmental outcomes

Children’s development was assessed using scores on the Chinese version of the ASQ, third edition (ASQ-3), completed by the parents or guardians of the children. The ASQ-3 is widely used to assess development in the communication, gross motor, fine motor, problem-solving, and personal-social domains. Each domain comprised 6 questions, and the total scores ranged from 0 to 60 points per domain, with higher scores indicating better development.^22^ The mean ASQ-3 scores of the 5 domains, or each domain, were used as continuous variables. A lack of useful vision in either eye was regarded as visual impairment, whereas the need for hearing aids in both ears was regarded as hearing impairment. A Palisano gross motor function score of ≥2 was defined as cerebral palsy per the Gross Motor Function Classification System.^23^ The diagnosis of autism spectrum disorders was based on parents’ reports.

#### Outcomes

The primary outcome was the long-term effects of ACBMNC infusions on neurodevelopmental outcomes in infants enrolled for up to 5 years. The primary endpoints were mean ASQ and ASQ-3 scores for each domain. Secondary endpoints were pneumonia-related hospitalization, oxygen use, wheezing episodes, and anthropometric parameters.

### Statistical analysis

Mean (standard deviation) and unpaired Student’s *t*-tests were used for continuous variables with normal distribution and median and interquartile range, respectively, while nonparametric analysis was used for data with non-normal distribution. Categorical variables were reported as numbers and percentages. Group comparisons of categorical variables for outcomes were performed using Fisher’s exact test or the chi-square test, as appropriate. Logistic and linear regressions were used to estimate the contribution of the intervention to outcomes after adjusting for gender, caregivers being parents, acquiring advanced education, and caregivers being smokers. All statistical tests were two-tailed, and statistical significance was set at *P* < .05. All statistical analyses were done using SPSS 21.0 (IBM).

## Results

### Study population

**Figure 1.**
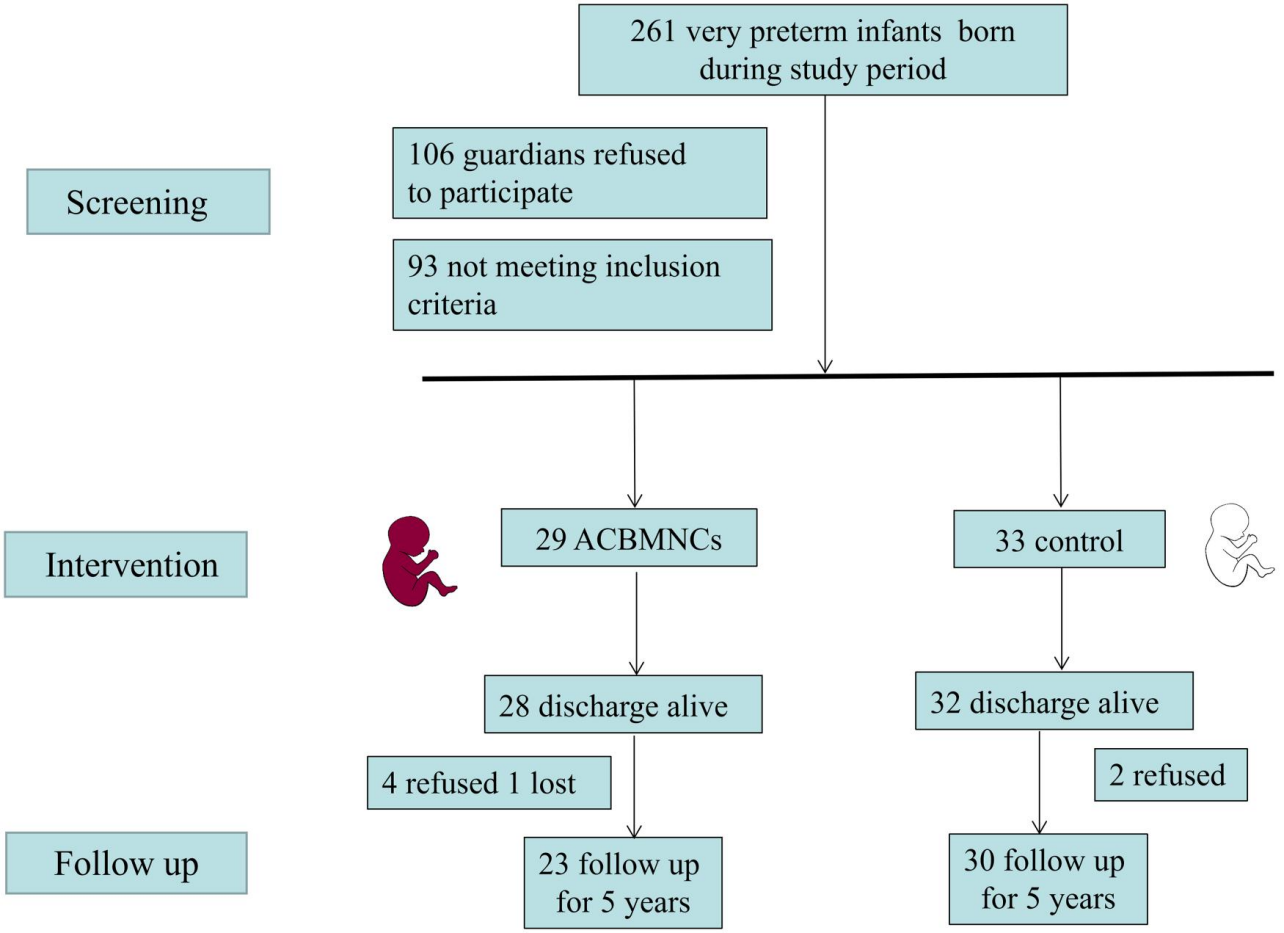
The design and process of this study. ACBMNC: autologous, cord blood mononuclear cells.

In the primary study, 62 infants were enrolled: 29 in the ACBMNCs intervention group and 33 in the placebo control group. Of the infants enrolled in the primary study (July 01, 2018, to January 1, 2020), 60 survived and were discharged.^20^ Two patients in the control group and 5 in the intervention group were lost in the 5-year follow-up study because of changing contact phone numbers; 5 of them were examined in a clinic and were healthy, and the other 2 patients were without severe complications when discharged home; therefore, we assumed that they were alive. Finally, 23 (82.14%) infants in the intervention group and 30 (93.75%) in the control group were followed up successfully (Figure 1).

### Baseline characteristics

The demographic and clinical characteristics of the study participants are presented in Table 1. No significant differences were observed in the baseline demographic and clinical characteristics between the groups. In the intervention group, there were more female infants, more caregivers being parents and acquiring advanced education, and fewer caregivers being smokers. These factors may affect both respiratory and neurodevelopmental outcomes; thus, they were adjusted in the outcome analysis (Table 1).

**Table 1.** Baseline characteristics of the patients.

Characteristics	Control group^24^	Intervention group^23^	*P* value
**Gestational age (weeks)**	29.72 ± 1.21	29.96 ± 1.40	.505
**GA < 28 weeks**	3/30,10%	2/23, 8.7%	1.000
**Birth weight (kg)**	1.34 ± 0.23	1.38 ± 0.34	.668
**Birth length (cm)**	37.76 ± 3.53	37.87 ± 3.63	.918
**Male sex**	16/30, 53.3%	7/23, 30.4%	.162
**Main caregiver-parents**	23/30, 76.7%	19/23, 82.6%	.738
**Main caregiver education, high school or advanced**	14/30, 44.2%	13/23, 55.8%	.583
**Main caregiver smoking**	7/30, 23.3%	3/23, 13%	.484
**Lost follow-up**	2/32, 6.3%	5/28, 17.9%	.235

Abbreviation: GA, gestational age.

For categorical variables, results are shown as no. /total no. (%). For continuous variables, results are shown as means and standard deviation.

### Neurodevelopmental outcomes

Neurodevelopmental outcomes included hearing impairment, visual impairment, cerebral palsy, seizures, infantile autism, and ASQ results. One infant in the control group was managed using bilateral hearing aids. One infant in each group presented with visual impairment until 5 years of age. In the control group, 2 infants had cerebral palsy, and 1 infant had cerebral palsy in the intervention group. One patient in the control group had infantile autism. There were no significant differences in the incidence of hearing impairment, visual impairment, cerebral palsy, seizure, or infantile autism between the groups until 5 years of age (Table 2).

**Table 2.** Neurodevelopmental outcomes.

Neurodevelopmental outcomes		Control group^24^	Intervention group^23^	*P* value	Adjusted *P* value
	Auditory impairment	1/30,3.3%	0/23,0%	1.000	
**Bayley score**	Visual impairment	1/30,3.3%	1/23,4.3%	1.000	
**ASQ** ^a^	Mean	51.38 ± 13.05	55.75 ± 3.70	.113	.081
	Communication	56.12 ± 16.11	59.00 ± 2.67	.280	.065
	Gross motor	52.12 ± 15.82	52.05 ± 13.91	.987	.975
	Fine motor	45.04 ± 17.18	53.41 ± 8.65	***.044***	***.038***
	Problem solving	53.27 ± 11.66	56.82 ± 4.24	.183	.067
	Personal-social	50.38 ± 16.67	57.50 ± 5.29	***.048***	***.044***
	Seizure	3/30,10%	1/23,4.3%	.624	
	Cerebral palsy	2/30,6.7%	1/23,4.3%	1.000	
	Infantile autism	1/30,3.3%	0,0%	1.000	

a Control group (*n* = 26), intervention group (*n* = 22). ASQ: Ages and Stages Questionnaires

For categorical variables, results are shown as no./total no. (%).

For continuous variables, results are shown as means and standard deviation. The bold and italic values mean *p* ＜0.05.

Twenty-six patients in the control group and 22 in the intervention group completed the questionnaire. Fine motor and personal-social domain scores were higher in the intervention group than in the control group (adjusted *P* = .038 and .044, respectively; Table 2). Otherwise, there were no significant differences between the mean ASQ scores and those of the other 3 domains.

**Figure 2.**
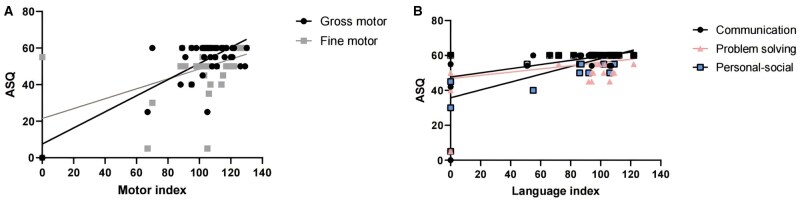
The correlation between Bayley scores in a 2-year follow-up and ASQ scores in a 5-year follow-up. (A) The motor index of Bayley score with scores of gross (*R*^2^ = 0.6407, *P* < .001) and fine motor (*R*^2^ = 0.2325, *P* = .0012) domains in ASQ; (B) The language index of the Bayley score with scores of communication (*R*^2^ = 0.2103, *P* = .0017), problem solving (*R*^2^ = 0.1237, *P* = .0019), and personal-social (*R*^2^ = 0.4252, *P* < .001) domains in ASQ.

There was a positive correlation between the motor index in the Bayley score at the 2-year follow-up and scores of gross (*R*^2^ = 0.6407, *P* < .001) and fine motor (*R*^2^ = 0.2325, *P* = .0012) domains (Figure 2A). The language index of the Bayley score also correlated positively with scores in the communication (*R*^2^ = 0.2103, *P* = .0017), problem-solving (*R*^2^ = 0.1237, *P* = .0019), and personal-social (*R*^2^ = 0.4252, *P* < .001) domains (Figure 2B).

### Anthropometric and respiratory outcomes

The general characteristics of the study infants during the follow-up assessment are shown in Table 3. No significant differences were found in body weight, height, or BMI. The percentages of patients with low body weight or short stature were similar between the two groups. No patient required supplemental oxygen. No pneumonia-related hospitalizations occurred during the latest year in either the control or intervention groups. Compared with the intervention group, more children in the control group had nocturnal cough (70% vs 30.4%, adjusted *P* = .021; Table 3).

**Table 3.** Anthropometric and respiratory outcomes.

Characteristics		Control group^24^	Intervention group^23^	*P* value	Adjusted *P* value
**Respiratory outcomes**	Wheezing	3/30, 10.0%	5/23, 21.7%	.272	.100
	Nocturnal cough	11/30, 70%	7/23, 30.4%	***.006***	***.021***
	Pneumonia related hospitalizations	0/30, 0%	0/23, 0%	1.000	1.000
	Supplemental oxygen	0/30, 0%	0/23, 0%	1.000	1.000
**Anthropometric characteristics**	*W* (kg)	16.93 ± 2.31	16.07 ± 1.73	.139	
	*W* ＜ 3%	6/29, 20.7%	4/23, 17.4%	1.000	
	*H* (cm)	108.73 ± 8.03	105.13 ± 6.25	.081	
	*H* ＜ 3%	9/30, 30.0%	5/23, 21.7%	.547	
	BMI (kg/m^2^)	14.35 ± 1.70	14.55 ± 1.18	.627	

Abbreviations: BMI, body mass index; *H*, height; *W*, weight.

For categorical variables, results are shown as no./total no. (%). For continuous variables, results are shown as means and standard deviation. The bold and italic values mean *p* ＜0.05.

## Discussion

In this study, we followed up the infants previously enrolled in a prospective, nonrandomized, placebo-controlled trial investigating the effect of ACBMNCs infusion soon after birth on severe BPD prevention for 5 years.^20^ During the 5-year follow-up period, we found that intravenously infused ACBMNCs significantly improved the scores of the fine motor and personal-social domains in the ASQ assessment, indicating a better neurodevelopmental outcome. Our previous study showed that an intravenous dose of ACBMNCs significantly decreased the incidence of moderate or severe BPD in surviving very preterm neonates.^20^ A 2-year follow-up outcome showed that the incidence of developmental delay and pneumonia-related hospitalizations was reduced in the intervention group.^20^ This study also showed high consistency between the motor index in the Bayley score in a 2-year follow-up with both gross and fine motor domain scores, as well as the language index with communication, problem-solving, and personal-social domain scores. Although pneumonia-related hospitalizations were similar between the two groups, the invention group showed less nocturnal cough.

BPD has been known to be a key contributor to the substantial healthcare burden associated with preterm birth.^1^ Worse, BPD is associated with a higher risk of mortality and long-term respiratory and neurological developmental retardation.^1^^,^^3^ Current therapies, such as lung-protective ventilation strategies, pulmonary surfactants, and steroid treatments, only alleviate the symptoms of BPD but do not reduce BPD severity or improve long-term outcomes.^1^^,^^2^ Corticosteroids have been used for several decades to prevent or treat BPD, as they may prevent the harmful effects of inflammation on the developing lung.^1^^,^^25^ However, several large RCTs have shown very limited improvement in BPD; in contrast, it may affect long-term neurodevelopmental outcomes.^26–28^ Several preclinical studies have reported beneficial effects of stem cell therapies in the prevention and treatment of BPD.^10^ A previous clinical study showed that ACBMNC infusion safely reduced the duration of respiratory support and reduced BPD severity.^20^ However, long-term outcomes of stem cell therapy have rarely been reported.

In numerous recent reports, patients with BPD manifested long-term neurodevelopmental impairments, including seizures, cerebral palsy, autism, and developmental delays.^3^^,^^18^ Previously, a descriptive study reported the 2-year safety outcomes of the first-in-human study of amniotic cells for bronchopulmonary dysplasia but without a comparison group.^29^ The results of a study investigating ACB infusion in young children with cerebral palsy suggested improved brain connectivity and gross motor function at 1-year follow-up.^24^ A Korean team performed a clinical trial aimed at preventing BPD by intratracheally instilling allogeneic MSCs in extremely preterm infants and reported no statistically significant difference in respiratory and neurodevelopmental outcomes between the two groups during a 5-year follow-up. In the GA 25-28 weeks subgroup, the MSC group showed a lower incidence of developmental delay than the control group in the motor function domain, which was not consistent with the original BPD prevention results.^19^ Our long-term follow-up study is the first to demonstrate that ACBMNCs infusion has a persistent protective effect on the neurological development and respiratory outcomes in preterm infants.

It is strongly and independently associated with several neurodevelopmental disabilities.^3^^,^^18^ A previous EPIPAGE-2 cohort study showed that motor skills and BPD were associated with developmental coordination disorders. In addition, behavioral difficulties are independently associated with BPD.^3^ The ASQ has proven to be a reliable and valid screening tool for predicting developmental delay and has been widely used as an international health screening program for neurodevelopmental disorders in infants and children.^30^ Our study observed higher ASQ scores in the fine motor domain, indicating that ACBMNC therapy may improve motor coordination. The personal-social domain scores also increased in the intervention group, demonstrating better behavioral outcomes. In children with BPD, fragility persists for several years after discharge from the neonatal care unit.^3^^,^^18^ Preventing neurodevelopmental disabilities is a major concern in preterm children. Previous studies on the long-term follow-up of stem cell therapy in neonates have not provided a temporal association.^30–32^ Long-term outcomes in preterm infants may advance with the attenuation of BPD severity.^1^^,^^3^ Our study showed a high consistency in developmental improvement between the Bayley scores in a 2-year follow-up and the ASQ scores in a 5-year follow-up, which indicated that the beneficial effect of ACBMNCs on neurodevelopment was stable and persisted with age. These findings have rarely been reported in the literature and underscore the importance of improving the medical and neurodevelopmental management of BPD in preterm children, thus reducing its long-term consequences.

The current study had some limitations. First, although the baseline demographic data did not show a significant difference between the two groups, we found that in the intervention group, there were more female infants, more caregivers being parents and acquiring advanced education, and fewer caregivers were smokers. These factors may affect neurodevelopmental and respiratory health. To avoid potential bias, the outcome data were adjusted for possible confounding factors. Second, the study was nonrandomized, and the sample size was too small to reliably assess the efficacy of long-term outcomes. In addition, the number of patients aged <28 weeks was limited (only 5 patients in total). Multicenter randomized trials are needed to confirm the effect of ACBMNCs on neurodevelopment in premature infants. Third, the neurodevelopmental follow-up outcomes were assessed mainly by parental interviews and questionnaires, which may introduce subjective bias. A more objective evaluation, such as the Wechsler Intelligence Scale for Children, should be conducted in follow-up studies.

In conclusion, intravenously infused ACBMNCs showed a high potential for improving the primary endpoint of neurodevelopmental outcomes in infants born very preterm during the 5-year follow-up. Further randomized placebo-controlled trials are essential to confirm these findings.

## Data Availability

The datasets used and/or analyzed during the current study are available from the corresponding author on reasonable request.
